# Evaluation of the Neutralizing Antibodies Response against 14 SARS-CoV-2 Variants in BNT162b2 Vaccinated Naïve and COVID-19 Positive Healthcare Workers from a Northern Italian Hospital

**DOI:** 10.3390/vaccines10050703

**Published:** 2022-04-29

**Authors:** Josè Camilla Sammartino, Irene Cassaniti, Alessandro Ferrari, Federica Giardina, Guglielmo Ferrari, Federica Zavaglio, Stefania Paolucci, Daniele Lilleri, Antonio Piralla, Fausto Baldanti, Elena Percivalle

**Affiliations:** 1 Molecular Virology Unit, Microbiology and Virology Department, Fondazione IRCCS Policlinico San Matteo, 27100 Pavia, Italy; jose.sammartino@iusspavia.it (J.C.S.); alessandro.ferrari04@universitadipavia.it (A.F.); federica.giardina01@universitadipavia.it (F.G.); guglielmo.ferrari01@universitadipavia.it (G.F.); f.zavaglio@smatteo.pv.it (F.Z.); s.paolucci@smatteo.pv.it (S.P.); d.lilleri@smatteo.pv.it (D.L.); a.piralla@smatteo.pv.it (A.P.); fausto.baldanti@unipv.it (F.B.); e.percivalle@smatteo.pv.it (E.P.); 2 Department of Clinical, Surgical, Diagnostics and Pediatric Sciences, University of Pavia, 27100 Pavia, Italy

**Keywords:** SARS-CoV-2, immune response, RNA vaccine, variants of concern

## Abstract

SARS-CoV-2 still represents a global health burden, causing more than six million deaths worldwide. Moreover, the emergence of new variants has posed new issues in terms of vaccine efficacy and immunogenicity. In this study, we aimed to evaluate the neutralizing antibody response against SARS-CoV-2 variants in different cohorts of vaccinated and unvaccinated subjects. Four-fold diluted sera from SARS-CoV-2 naïve and recovered subjects vaccinated with two or three doses of the BNT162b2 vaccine were challenged against 14 SARS-CoV-2 variants, and the SARS-CoV-2 neutralizing antibody titer was measured. Results were compared with those obtained from unvaccinated COVID-19 recovered patients. Overall, a better SARS-CoV-2 NT Abs response was observed in recovered vaccinated subjects after three doses of the vaccine when compared to unvaccinated patients and vaccinated subjects with only two doses. Additionally, the lowest level of response was observed against the Omicron variant. In conclusion, third doses of BNT162b2 vaccine seems to elicit a sustained response against the large majority of variants.

## 1. Introduction

Since the beginning of the Coronavirus infectious disease 2019 (COVID-19) pandemic in March 2020 [1] caused by the severe acute respiratory syndrome coronavirus 2 (SARS-CoV-2), there have been 508,827,830confirmed cases and 6,227,291deaths worldwide [2]. SARS-CoV-2 is a novel human coronavirus first reported in China in December 2019 which spread worldwide with over 500 million confirmed cases that led the WHO to declare a state of pandemic in March 2020 [2]. The rapid spread of the disease has prompted intense research activity to identify potential treatments, including investigations on existing drugs and the parallel de novo development of innovative treatments. In January 2020, the Chinese Center for Disease Control and Prevention released the genetic sequence of the first SARS-CoV-2 isolated in Wuhan, China, which led to the development of the BNT162b2 vaccine [3]. BNT162b2 is a novel RNA-based vaccine encoding the full-length SARS-CoV-2 Spike protein, with a 95% protection rate against COVID-19, approved for emergency use by the WHO in December 2020, only 11 months after its development started [4]. The rapidity of approval reflects the seriousness of the current situation [5]. The availability of a vaccine is particularly important in inducing neutralizing humoral and cellular immunity and, more importantly, reducing COVID-19 infections, hospitalizations, and deaths in clinical trials. Further, in a hospital context, a vaccine is important for healthcare workers, not only for their own safety while working on the front line, but also to protect the patients from the spread of the disease [6]. Moreover, the development of mutations in the Spike sequence, which codes for the principal antigenic target of the new vaccines, leads to the insurgence of new variants. This raises concern over the neutralizing activity of vaccine-induced antibody responses, and the ability of antibodies triggered by previous infection to protect against re-infection [7], thus evaluating the BNT162b2 immunogenicity is fundamental. The Omicron variant, first identified in Botswana and South Africa, and which started spreading in November 2021, consists of over 30 mutations within the Spike protein and represents the last variant that can trigger the effect of the vaccines. In this context, we aimed to assess the humoral immune response elicited by the BNT162b2 vaccination in naïve and previously COVID-19 positive healthcare workers from the Fondazione IRCCS Policlinico San Matteo of Pavia, after two and three doses of vaccine, in comparison with a historical cohort of COVID-19 convalescent unvaccinated plasma donors. We determined the neutralizing titers of sera from all subjects of the study against the original virus (Wuhan, A), the Italian reference strain (D614G, B.1) and an additional 12 different variants of SARS-CoV-2, including the main variants of concern (VOC) with particular focus on the Omicron variant.

## 2. Materials and Methods

### 2.1. Study Population

All subjects included in the study were presented with an informed written consent prior to the sampling of the sera. An overview of the test population is reported in Table 1.

#### 2.1.1. COVID-19 Convalescent Plasma Donors (PD) Cohort Characteristics

The cohort is composed of 30 subjects who did not receive BTN162b2 vaccination and had a previously reported history of COVID-19 positivity. In the group, there are 80% male and 20% female subjects with a median age of 67 years (range 35–84). All presented mild common symptoms, and none needed to be hospitalized. The sera were collected during the first wave of COVID-19 infections between May and July 2020, at 26.4 days on average after the onset of symptoms (median days 17; range 12–103).

#### 2.1.2. Naïve Healthcare Workers (Naïve HCW) Cohort Characteristics

The cohort is composed of 30 subjects who did not have a reported history of COVID-19 and tested negative for antibodies against the SARS-CoV-2 Nucleoprotein. The group consists of 20% male and 80% female subjects with a median age of 52 years (range 30–66). Eight subjects were excluded from the follow-ups.

#### 2.1.3. COVID-19 Exposed Healthcare Workers (Exposed-HCW) Cohort Characteristics

The cohort is composed of 16 subjects who did receive at least two doses of the BTN162b2 vaccination at enrollment and had a previously reported history of COVID-19 positivity. In the group, there are 13% male and 87% female subjects with a median age of 42 years (range 25–61). In all, 14 subjects presented mild common symptoms, while 2 had none. No subject of the group needed hospitalization. During follow-ups, two subjects did not receive the third dose of vaccine and four did not participate in the follow-up analysis, thus being excluded.

#### 2.1.4. Omicron Healthcare Workers (Omicron-HCW) Cohort Characteristics

The cohort is composed of 15 subjects who received three doses of the BTN162b2 vaccination but got infected with the Omicron variant during an Omicron outbreak in one of the Policlinico San Matteo departments. There are a 54% male and 46% female subjects with a median age of 28 (range 26–34). In all, 12 subjects had mild symptoms, with only one reporting dyspnea; none needed to be hospitalized. The symptoms lasted a mean of 7.7 days (median: 6; range 2–20). The first positivity was reported on average after 54.8 days (median: 71; range 2–75) after the 3rd dose and lasted for about 14.7 days (median: 15; range 7–19). One subject had reported history of COVID-19 (infected with the Delta variant) at 7 months from the 2nd dose. The sera were sampled on average 25.6 days after the first positive molecular test (median: 27; range 18–30).

### 2.2. Virus Isolation

All variants were isolated from 200 uL of nasopharyngeal swabs seeded on confluent VERO E6 cells (VERO C1008 (Vero 76, cloneE6, Vero E6); ATCC^®^ CRL-1586™) in a 24-well flat-bottom tissue-culture microtiter plate (COSTAR, Corning Incorporated, Corning, NY, USA) decontaminated and incubated at 33 °C with 5% CO_2_ for 1 h. After inoculum removal, fresh MEM eagle (EMEM, Lonza Group Ltd., Basel, Switzerland) supplemented with 1% *v/v* Penicillin, Streptomycin, Glutamine (Euroclone SpA) and 0.1% *v/v* Trypsin was added before incubation, in the same conditions, until cytopathic effect development. All the samples inoculated were observed under an inverted microscope, 10× magnification, every other day, until cytopathic effect (CPE) development was observed. SARS-CoV-2 CPE on VERO E6 cells is characterized by cell enlargement and syncytia formation. After the first isolation, all the variants were propagated in a VERO E6 25 cm^2^ cell culture flask (Corning Incorporated, Corning, NY, USA) to increase virus titer and to prepare virus stock for microneutralization testing.

### 2.3. Whole Genome Sequencing

All variants were confirmed through complete genome sequencing [8] in order to confirm the presence of variant-defining mutations, and sequences were submitted to the Global Initiative on Sharing Avian Influenza Data (GISAID).

### 2.4. Virus Titration and Microneutralization Test

The titer of each variant’s stock was measured at the 50% tissue culture infectious dose (TCID50) in six replicas in a 96-well flat-bottom tissue-culture microtiter plate. Briefly, logarithmic dilutions of previously stocked virus in presence of 3 × 10^4^ VERO E6 cells were incubated for 72 h at 33 °C in 5% CO_2_. The cells were observed under a microscope for cytopathic effect development and stained with Gram’s crystal violet solution (Merck KGaA, Darmstadt, Germany) plus 5% *v/v* formaldehyde 40% m/v (Carlo Erba SpA, Arese, Italy). The value of TCID50 mL^−1^ was calculated with the Reed–Muench method [9]. After virus titration, 50 µL of 100 TCID50 was incubated with 50 µL of serial dilutions (1:10 to 1:640) of the subject’s sera in duplicate in a 96-well flat-bottom tissue-culture microtiter plate. After 1 h incubation at 33 °C in 5% CO_2_, 3 × 10^4^ VERO E6 cells were added to each well. After 72 h incubation, wells were stained with Gram’s crystal violet solution as previously reported. The neutralizing titer demonstrated the maximum dilution with the reduction of 90% of cytopathic effect. A positive titer was equal to or greater than 1:10 [7,8].

### 2.5. Statistical Analysis

Comparison between groups was performed using two-way analysis of variance (ANOVA) with post hoc Dunnett’s correction. GraphPad Prism 8.3.0 (GraphPad Software, La Jolla, CA, USA) was used for statistical analyses. A two-sided *p* value < 0.05 is considered statistically significant.

## 3. Results

### 3.1. Viral Isolation and Characterization

**Figure 1:**
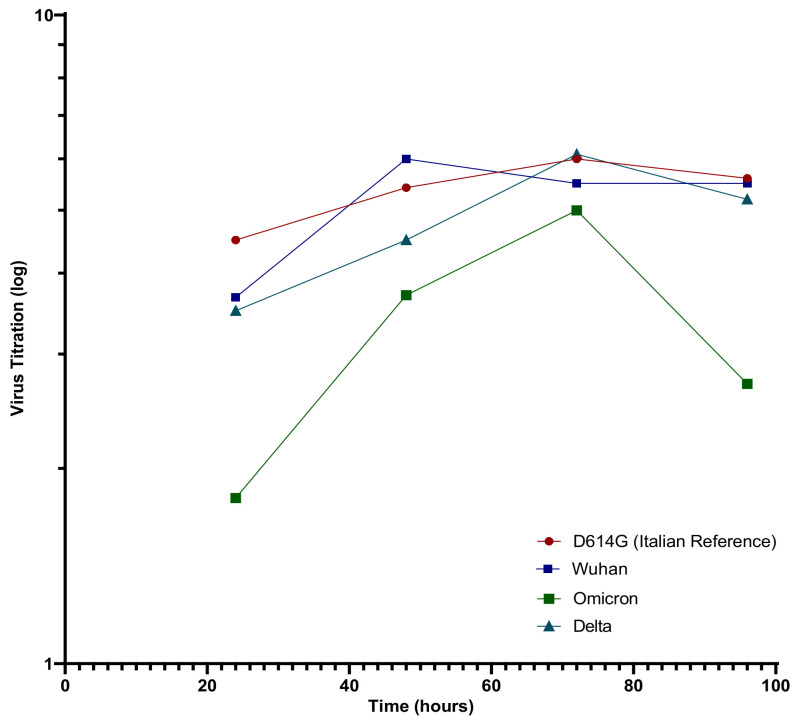
SARS-CoV-2 variants titration curves.

SARS-CoV-2 variants were isolated from nasopharyngeal swabs, and whole genome sequencing was performed (Table 2). All the variants were titrated and the growth curves for the Wuhan, D614G, Delta (B.1.617.2), and Omicron (B.1.1529; BA.1) strains were determined (Figure 1). We observed that the Wuhan strain grew faster, reaching the growth peak at 48 h and slowly decreasing afterwards. On the other hand, D614G and Delta both peaked at 72 h, followed by the Omicron variant that grew slowly. In detail, The Wuhan and Delta variant titers were 1.0 ± 0.2 log lower in comparison to the D614G values at each time point but had the same trend. The overall trend of the Omicron growth curve mimics the other variants but is slightly translated to the right, growing at a lower level than the other VOC. In particular, Omicron had a titer 2.5 log lower at 24 h, and a 1.4 log lower at 48 h in comparison to D614G values. At 72 h, there was a slight recovery in the growth speed, with a titer of only a 1.2 log lower in comparison to D614G but was almost identical to the Wuhan and Delta values at the same time points. At 96 h, the Omicron variant showed a drop, with titer values log 2 that were lower than those of D614G.

### 3.2. Assessing Antibody Response through Microneutralization Test

**Figure 2:**
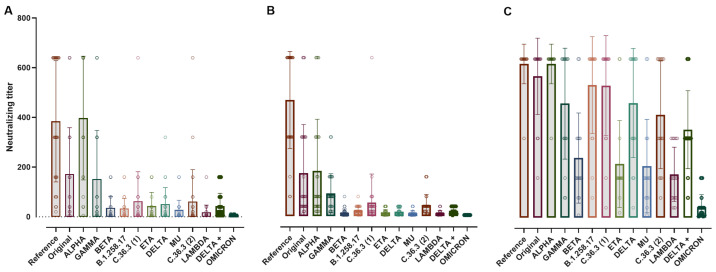
Scatter dot plot of the neutralizing titer for convalescent-PD (**A**), naïve-HCW (**B**) and exposed-HCW (**C**) following 2 doses of BTN162b2. In the graph, the bars stop at the titer mean for each variant. The convalescent-PD group has a high response only for the reference strain while the naïve-HCW has a high response to both the reference and alpha variants, but overall mimics the convalescent-PD trend. The experienced-HCW cohort achieved the highest response to all the different SARS-CoV-2 variants between the groups. High titers were associated with a larger variability (higher SD). Reference: D614G strain; Original: Wuhan.

At 1 month post-2nd dose, SARS-CoV-2 NT-Abs in naïve healthcare workers (HCW) and exposed-HCW were compared to results obtained in unvaccinated COVID-19 convalescent plasma donors (PD) (Figure 2).

The highest response levels were observed in vaccinated COVID-19 exposed-HCW (Figure 2C), SARS-CoV-2 NT-Ab titers were lower in COVID-19 convalescent-PD (Figure 2A) and naïve-HCW (Figure 2B). The convalescent-PD group had the higher response to Alpha and D614G strains, followed by Wuhan and Gamma strains, while the results obtained with other variants scored between 1:20 and 1:80, except for Lambda and Omicron variants, which were lower. The naïve-HCW had the highest response to D614G, followed by Alpha and Wuhan, while the response to the other variants was lower, with Delta, Eta, Beta, Lambda, Mu, and Omicron values below 1:20. The exposed-HCW group had the strongest response in comparison to the other groups, with NT-Ab against D614G and Alpha scoring the highest followed by Wuhan, B.1.258.17. C.36.3 (1), Delta and Gamma, with values ranging 1:460 to 1:620, while for C.36.3 (2), Delta+ (1:400 and 1:350 respectively), Beta, Eta, Mu, Lambda, and Omicron (in range 1:40 to 1:250), there was a significant reduction in the NT-Ab response in comparison to the response to D614G (Figure 2). Detailed data on the NT-Ab response can be found in the Supplementary Table S1.

**Figure 3:**
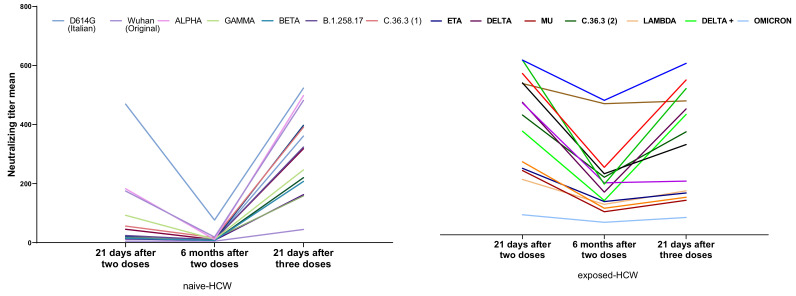
Data show the progression of the neutralizing antibody titer (NT-Ab) in the naïve HCW (**left**) and exposed-HCW cohorts (**right**). In both groups, there was a reduction in the NT-Ab at 6 months post-2nd dose, but the titer was recovered at 1 month after the 3rd dose.

The kinetics of the neutralizing antibody titer (NT-Ab) in the naïve-HCW and exposed-HCW cohorts following two doses (1- and 6-month follow-up) and three doses of BTN162b2 vaccination is reported in Figure 3.

As expected, at 6 months post-2nd dose, the overall NT-Abs response decreased, with a 65% mean reduction for the naïve HCW and 55% for the exposed-HCW, which, however, maintained higher values. The NT-Ab against the D614G variant had the highest reduction at 6 months and the least recovery after the 3rd dose in the naïve-HCW cohort, which had the opposite trend in the exposed-HCW. Alpha had the highest reduction at 6 months in both groups, while Delta and Delta+ had the highest recovery at 1 month post-3rd dose. After three doses, the D614G variant had the overall higher response in all cohorts, while Omicron had the lowest (Figure 3).

**Figure 4:**
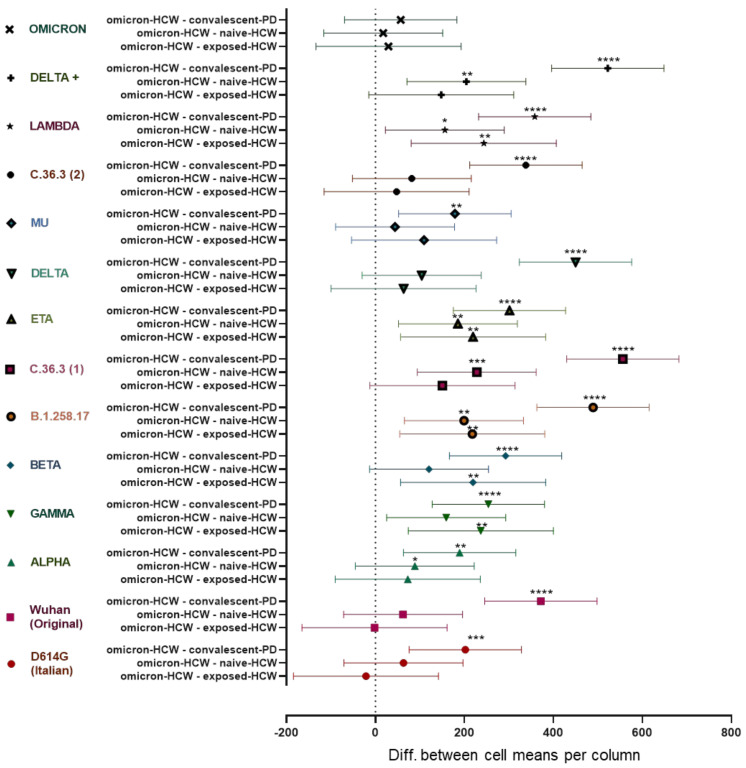
Data show the difference in means of the neutralizing titer (NT-Ab) against 14 different SARS-CoV-2 variants in naïve-HCW and exposed-HCW after 3 doses of BTN162b2 and in unvaccinated convalescent-PD, in comparison to the fully vaccinated Omicron-HCW NT-Ab values. The more distant the values are from the origin line (0), the higher the difference between the paired populations. **** *p* < 0.0001; *** *p* = 0.0001; ** *p*= 0.001; * *p* < 0.05. (Omicron-HCW—convalescent-PD: mean difference between Omicron-HCW and convalescent-PD groups; Omicron-HCW—naïve-HCW: mean difference between Omicron-HCW and naïve-HCW groups; Omicron-HCW—experienced-HCW: mean difference between Omicron-HCW and experienced-HCW groups).

Data obtained from a group of triple dosed vaccinated HCW with subsequent exposure to the Omicron variant (Omicron-HCW) were compared to data obtained by the other groups of the study to investigate how the NT-Ab response changes in relation to the infection with a different, more recent variant of SARS-CoV-2 especially as the infection occurred after the full course of vaccinations was administered (Figure 4).

The highest difference was observed between the convalescent-PD and Omicron-HCW groups. On average, the convalescent-PD NT-Ab values were 8-fold lower than the Omicron-HCW with the highest differences observed for the Lambda (20-fold), B.1.258.17 (15-fold), Beta, Delta, and C.36.3 (1) (9-fold each) variants. Differently, the naïve-HCW and exposed-HCW had similar responses against the variants of SARS-CoV-2, thus scoring similarly to the Omicron-HCW, with average NT-Ab values respectively 1.4 and 1.7 times lower than the Omicron-HCW. The smallest significative differences between groups were observed in response to the D614G and the Alpha variants, while there was no significative difference in the response to the Omicron variant between the groups of the study (Figure 4).

In particular, the convalescent-PD cohort responded with titers higher than 1:300 only to the D614G and the Alpha variants, while both naïve-HCW and exposed-HCW had titers higher than 1:300 for 8 of the 14 variants tested after 3rd dose administration. Overall, the Omicron-HCW had the best response, with a NT-Ab titer higher than 1:300 for 12 of the 14 variants tested. Interestingly, the lower level of SARS-CoV-2 NT-Abs response was observed against the Omicron variant in all of the study cohorts with median titers ranging from negative to 1:60 (Table 3; Table S1).

## 4. Discussion

Vaccination with new mRNA vaccines can stimulate a substantial humoral response with the production of neutralizing antibodies highly specific for the defined antigens. On the other hand, thousands of COVID-19 variants were detected all over the world and few of them are still considered a concern [12]. Coronaviruses, including SARS-CoV-2, develop many mutations that are often not detrimental for their biological behavior and their structures [13]. It is conceivable that the variants would not undermine vaccine effectiveness in preventing severe COVID-19 but can have a role in reducing the vaccine immunogenicity. In particular, the rapid emergence of the new Omicron variant at the end of 2021, with more than 30 mutations at the Spike sequence, in the background of high Beta immunity, implies that the virus may have evolved to escape neutralization in Beta-specific serum raising new issues in terms of vaccine efficacy and the use of monoclonal antibodies in clinical practice. In agreement with our results, it has been observed that in two dosed vaccinated subjects SARS-CoV-2 NT-Ab levels were reduced against Omicron but improved when the third dose of vaccine was administered [14,15]. Interestingly, we observed as other authors did, a reduced replication capacity on permissive VERO E6 cells of the Omicron variant in comparison to the reference strain and Delta variant. Differences in the morphology of infected cells between Omicron and Delta were also observed, suggesting that Omicron is less fusogenic than Delta [16]. These data are confirmed also by the clinical features of infection characterized by an attenuated pathogenicity.

Based on our results, two doses of BNT162b2 vaccine are able to elicit an antibody response in naïve subjects against variants harboring the sequence used for vaccine construction, while in previously infected subjects the response is broader, covering different SARS-CoV-2 variants, as previously reported [17]. An overall reduction in SARS-CoV-2 NT-Abs against each variant tested was observed at six months after vaccination, as expected. However, after the third dose, the NT-Ab response was recovered.

As the convalescent-PD cohort was sampled before the start of the vaccination campaign, when there were few circulating variants, the presence of antibodies against different newer strains shows how natural infection provides wider coverage maintaining a reasonable effectiveness also against the new Omicron variant. So far, a humoral and a potent cell-mediated response, which are involved in protection against severe disease in COVID-19 patients, was observed for at least 15 months after the onset of symptoms, suggesting a long-term response elicited by natural infection [18]. Nonetheless, vaccinated subjects infected with the newer Omicron variant show the highest response against all previously characterized strains of SARS-CoV-2, which is in accordance with previously published data [19]. However, Omicron can escape NT-Abs, causing milder symptoms, which can be attributed to its reduced fitness in the lower airways [20] and reduced replication in comparison to the other variants. Moreover, as stated by other authors, the antigenic profile of the Omicron receptor binding domain (RBD) is different from previous VOC, giving a reduced antigenicity in its new receptor binding sites (RBS) [21]. This evolutionary trend of decreasing antigenicity was also described for the old circulating coronavirus hCoV229E [21], which could confirm this hypothesis for the Omicron variant as well.

## 5. Conclusions

Altogether, these data show how important it is to vaccinate naïve subjects, but also that vaccination is helpful for previously infected subjects as it boosts their immune system and helps to keep the NT-Ab high in time. Moreover, as demonstrated by the high level of response in the Omicron-HCW cohort, boosting the immune system with different, newer variants could be beneficial in terms of antibody coverage. These findings are strengthened by the use of wild type isolated viruses better mimicking what could happen in a realistic setting. Therefore, it could be reasonable to suggest boosting vaccinated subjects with vaccines derived from circulating variants to implement immunity stimulation and ensure a broader coverage of immunity.

## Figures and Tables

**Table 1:** Overview of the study populations. For the experienced-HCW and the Omicron-HCW populations all the available subjects were included, while the groups convalescent-PD and naïve-HCW were randomly selected from the pool of plasma donors and healthcare workers of Policlinico San Matteo, respectively.

	*n*	Drop-Outs	COVID-19	Times Assayed	Vaccination
Convalescent-PD	30	none	yes	Pre-vaccination	no
Naive-HCW	30	8	no	1 month after 2nd dose; 6 months after 2nd dose; 1 month after 3rd dose	full course
Experienced-HCW	16	6	yes		full course
Omicron-HCW	15	none	yes	1 month after COVID-19 positivity post full course vaccination	full course

**Table 2:** Overview of the SARS-CoV-2 variants used in the study, reported with their corresponding mutations, the date of isolation, the lineage following Pangolin [10] and, where applicable, the WHO nomenclature [11].

Strain Name	WHO Nomenclature	Lineage (Pangolin)	Spike Mutations	Spike Deletions	GISIAD
hCoV-19/Italy/LAZ-INMI1-isl/2020	-	A	N679S	-	EPI_ISL_410545
hCoV-19/Italy/LOM-INMI-10734/2020	-	B.1	S247R, D614G	-	EPI_ISL_568579
hCoV-19/Italy/LOM-Pavia-10833/2020	Alpha	B.1.1.7	N501Y, A570D, D614G, P681H, R685H, T716I, S982A, D1118H	69–70, 144	EPI_ISL_7043618
hCoV-19/Italy/LOM-Pavia-10858/2021	Gamma	P1	L18F, T20N, P26S, D138Y, R190S, K417T, E484K, N501Y, D614G, H655Y, T1027I, V1176F	-	EPI_ISL_7043637
hCoV-19/Italy/LOM-Pavia-10860/2021	Beta	B.1.351	S171L, D80A, D215G, L242H, K417N, E484K, N501Y, D614G, A701V	243–245	EPI_ISL_7043650
hCoV-19/Italy/LOM-Pavia-10870/2021	-	B.1.258.17	L189F, N439K, D614G, V772I	69–70	EPI_ISL_7043668
hCoV-19/Italy/LOM-Pavia-10881/2021		C.36.3(1)	S12F, W152R, R346S, L452R, T547I, D614G, Q677H, A899S	69–70	EPI_ISL_7043684
hCoV-19/Italy/LOM-Pavia-10882/2021	Eta	B.1.525	Q52R, A67V, E484K, D614G, Q677H, F888L	69–70, 144	EPI_ISL_7043697
hCoV-19/Italy/LOM-Pavia-10916/2021	Delta	B.1.617.2	T19R, G142D, E156G, A222V, L452R, T478K, D614G, P681R, R682W, D950N, E990A	157–158	EPI_ISL_7043718
hCoV-19/Italy/LOM-Pavia-10919/2021	Mu	B.1.621.1	T95I, Y144T, R346K, N501Y, D614G, P681H, D950N	-	EPI_ISL_7462685
hCoV-19/Italy/LOM-Pavia-10921/2021	-	C.36.3(2)	S12F, W152R, R346S, L452R, D614G, A899S	69–70, 675–679	EPI_ISL_7043733
hCoV-19/Italy/LOM-Pavia-10924/2021	Lambda	C.37	G75V, T76I, R246N, L452Q, D614G, T859N	247–253, 675–679	EPI_ISL_7043746
hCoV-19/Italy/LOM-Pavia-10940/2021	DeltaPlus	AY.4.2.3	T19R, T95I, G142D, Y145H, R158G, A222V, L452R, T478K, D614G, P681R, D950N	156–157	Submitted
hCoV-19/Italy/LOM-Pavia-10943/2021	Omicron	B.1.1.529 (BA.1)	A67V, T95I, Y145D, L212I, G339D, S371L, S373P, S375F, N440K, G446S, S477N, T478K, E484A, Q493R, G496S, Q498R, N501Y, Y505H, T547K, D614G, H655Y, N679K, P681H, N764K, D796Y, N856K, Q954H, N969K, L981	69–70, 142–144, 211	Submitted

**Table 3:** Neutralizing titers of the different population of the study against the 14 SARS-CoV-2 variants tested. The results are expressed as means and standard deviations.

	Convalescent-PD		Naive-HCW		Exposed-HCW		Omicron-HCW	
Variant	Mean	SD	Mean	SD	Mean	SD	Mean	SD
D614G	384.7	244.1	523.6	157.6	608	101.2	586.7	144
Wuhan	172.3	186.3	481.8	205.3	546	210.4	544	168.9
Alpha	397.5	249.2	498.2	200.5	514	218.7	586.7	144
Gamma	151.7	196.4	246.4	230.7	168.5	94.81	405.4	240.8
Beta	35.67	46.75	207.5	220.5	108.5	58.79	328	215
B.1.258.17	33.5	40.81	323.6	215.6	305	242.5	522.7	176
C.36.3 (1)	62.67	119.1	390.9	226.3	468	235.6	618.7	82.62
Eta	42.83	55.83	158.6	164	124.5	59.84	344	229.2
Delta	51.5	65.8	397.3	222.6	438	272.7	501.3	180.1
Mu	27.83	38.9	162.3	201.9	97	58.51	206.4	189.4
C.36.3 (2)	61.83	128.2	318.2	225.4	352.5	222.9	400	215.9
Lambda	18	29.14	220	217.3	132.5	58.56	376	235.1
Delta +	43.17	51.55	360.9	245.2	417.5	260.8	565.3	158.5
Omicron	5.5	1.526	44.77	50.01	33	22.01	62.4	61.9

## Data Availability

Data available on request due to restrictions (privacy and ethical).

## Supplementary Materials

The following supporting information can be downloaded at: www.mdpi.com/article/10.3390/vaccines10050703/s1, Table S1: Descriptive statistics of the data analyzed in the study.
