# Supplementary material for: Evaluation of the Neutralizing Antibodies Response against 14 SARS-CoV-2 Variants in BNT162b2 Vaccinated Naïve and COVID-19 Positive Healthcare Workers from a Northern Italian Hospital
Source: Vaccines (Basel). 2022 Apr 29;10(5):703. doi: 10.3390/vaccines10050703 (PMC9145000; doi:10.3390/vaccines10050703)
Supplement: Supplementary file 1 [file vaccines-10-00703-s001.zip › vaccines-1678239-supplementary.pdf]

vaccines

MDPI

Supplementary Materials

Table S1. Descriptive statistics of the data analysed in the study.

		Reference	Original	ALPHA	GAMMA	BETA	B.1.258.17	A.36.3 (1)	ETA	DELTA	MU	C.36.3 (2)	LAMBDA	DELTA +	OMICRON
convalescent-PD	Number of values	30	30	30	30	30	30	30	30	30	30	30	30	30	30
	Minimum	20	5	5	5	5	5	5	5	5	5	5	5	5	5
	Maximum	640	640	640	640	160	160	640	160	320	160	640	160	160	10
	Range	620	635	635	635	155	155	635	155	315	155	635	155	155	5
	Mean	384,7	172,3	397,5	151,7	35,67	33,5	62,67	42,83	51,5	27,83	61,83	18	43,17	5,5
	Std. Deviation	244,1	186,3	249,2	196,4	46,75	40,81	119,1	55,83	65,8	38,9	128,2	29,14	51,55	1,526
	Std. Error of Mean	44,56	34,02	45,49	35,85	8,536	7,452	21,75	10,19	12,01	7,102	23,4	5,321	9,412	0,2785
naive-HCW 2-1	Number of values	30	30	30	30	30	30	30	30	30	30	30	30	30	30
	Minimum	80	10	20	10	5	5	5	5	5	5	10	5	4	5
	Maximum	640	640	640	320	80	80	640	40	40	40	160	40	40	5
	Range	560	630	620	310	75	75	635	35	35	35	150	35	36	0
	Mean	469,3	175	182,7	92,67	14,67	24	56,17	16,17	19,17	12,5	45,33	13,5	22,13	5
	Std. Deviation	195,7	195,4	208,8	79	15,25	16,05	114,6	11,87	13,46	9,978	42,73	9,662	13,54	0
	Std. Error of Mean	35,72	35,68	38,13	14,42	2,785	2,93	20,92	2,167	2,457	1,822	7,801	1,764	2,473	0
naive-HCW 2-6	Number of values	28	28	28	28	28	28	28	28	28	28	28	28	28	28
	Minimum	10	5	5	5	5	5	5	5	5	5	5	5	5	5
	Maximum	320	40	56	40	20	40	40	120	20	20	40	10	20	5
	Range	310	35	51	35	15	35	35	115	15	15	35	5	15	0
	Mean	76,43	19,11	10,93	11,61	7,679	9,643	16,79	12,93	8,571	8,036	10,54	7,5	8,036	5
	Std. Deviation	96,39	12,25	9,892	11,06	5,354	7,192	12,26	22,87	5,245	3,426	7,618	2,546	4,159	0
	Std. Error of Mean	18,22	2,315	1,869	2,09	1,012	1,359	2,318	4,322	0,9913	0,648	1,44	0,4811	0,7859	0
naive-HCW 3-1	Number of values	22	22	22	22	22	22	22	22	22	22	22	22	22	22
	Minimum	320	40	80	20	5	40	40	10	20	10	40	40	20	5
	Maximum	640	640	640	640	640	640	640	640	640	640	640	640	640	160
	Range	320	600	560	620	635	600	600	630	620	630	600	600	620	155
	Mean	523,6	481,8	498,2	246,4	207,5	323,6	390,9	158,6	397,3	162,3	318,2	220	360,9	44,77
	Std. Deviation	157,6	205,3	200,5	230,7	220,5	215,6	226,3	164	222,6	201,9	225,4	217,3	245,2	50,01
	Std. Error of Mean	33,59	43,76	42,74	49,19	47,01	45,96	48,24	34,97	47,45	43,04	48,06	46,32	52,28	10,66
exposed-HCW 2-1	Number of values	16	16	16	16	16	16	16	16	16	16	16	16	16	16
	Minimum	320	160	320	80	10	80	40	20	40	10	80	40	80	5
	Maximum	640	640	640	640	640	640	640	640	640	640	640	320	640	160
	Range	320	480	320	560	630	560	600	620	600	630	560	280	560	155
	Mean	620	570	620	460	240,6	535	532,5	216,3	462,5	208,1	415	175	355	43,13
	Std. Deviation	80	154,2	80	223,4	181,6	195,3	201,7	175,5	220	188,2	217,1	109,2	157,2	49,73
	Std. Error of Mean	20	38,56	20	55,86	45,39	48,84	50,43	43,86	55,01	47,04	54,27	27,29	39,31	12,43
exposed-HCW 2-6	Number of values	16	16	16	16	16	16	16	16	16	16	16	16	16	16
	Minimum	80	40	40	40	10	20	40	20	20	10	10	10	10	5
	Maximum	640	640	640	640	160	640	640	320	320	160	640	160	320	40
	Range	560	600	600	600	150	620	600	300	300	150	630	150	310	35

	**Mean**	470	220	157,5	162,5	67,5	196,3	457,5	92,5	127,5	54,38	183,1	81,88	96,25	15
	**Std. Deviation**	212,4	158	157,6	166,2	58,71	187,4	249,6	80,62	92,34	53,79	165,2	64,31	79,66	13,66
	**Std. Error of Mean**	53,1	39,5	39,41	41,55	14,68	46,84	62,39	20,16	23,08	13,45	41,29	16,08	19,91	3,416
**exposed-HCW 3-1**	**Number of values**	10	10	10	10	10	10	10	10	10	10	10	10	10	10
	**Minimum**	320	20	20	5	5	10	40	5	20	10	5	5	5	5
	**Maximum**	640	640	640	320	160	640	640	160	640	160	640	160	640	80
	**Range**	320	620	620	315	155	630	600	155	620	150	635	155	635	75
	**Mean**	608	546	514	168,5	108,5	305	468	124,5	438	97	352,5	132,5	417,5	33
	**Std. Deviation**	101,2	210,4	218,7	94,81	58,79	242,5	235,6	59,84	272,7	58,51	222,9	58,56	260,8	22,01
	**Std. Error of Mean**	32	66,54	69,16	29,98	18,59	76,67	74,49	18,92	86,23	18,5	70,48	18,52	82,48	6,96
**omicron-HCW 3**	**Number of values**	15	15	15	15	15	15	15	15	15	15	15	15	15	15
	**Minimum**	160	160	160	0,32	40	160	320	40	160	16	80	40	160	10
	**Maximum**	640	640	640	640	640	640	640	640	640	640	640	640	640	160
	**Range**	480	480	480	639,7	600	480	320	600	480	624	560	600	480	150
	**Mean**	586,7	544	586,7	405,4	328	522,7	618,7	344	501,3	206,4	400	376	565,3	62,4
	**Std. Deviation**	144	168,9	144	240,8	215	176	82,62	229,2	180,1	189,4	215,9	235,1	158,5	61,9
	**Std. Error of Mean**	37,17	43,61	37,17	62,19	55,51	45,43	21,33	59,18	46,49	48,89	55,75	60,7	40,92	15,98

Convalescent-PD: Covid-19 Convalescent Plasma Donors; naïve-HCW 2-1: Naive HealthCare Workers with 2 doses of BTN162b2 at 1-month follow-up; exposed-HCW 2-1: COVID-19 HealthCare Workers with 2 doses of BTN162b2 at 1-month follow-up; naïve-HCW 2-6: Naïve HealthCare Workers with 2 doses of BTN162b2 at 6-month follow-up; exposed-HCW 2-6: COVID-19 HealthCare Workers with 2 doses of BTN162b2 at 6-month follow-up; naïve-HCW 3-1: Naive HealthCare Workers with 3 doses of BTN162b2 at 1-month follow-up; exposed-HCW 3-1: COVID-19 HealthCare Workers with 3 doses of BTN162b2 at 1-month follow-up; omicron-HCW 3: Omicron HealthCare workers with 3 doses of BTN162b2 after omicron infection.
